# A Pharyngeal Pouch

**Published:** 1901-05-25

**Authors:** 


### A PHARYNGEAL POUCH.

At the last meeting of the Royal Medical and Chirurgical Society a case was related by Messrs. Rickman Godlee and Bucknall, in which a large pharyngeal pouch was removed by operation. On coming under observation in May, 1900, the patient had a swelling which looked exactly like an enlarged gland on the left side of the neck, at the level of the hyoid bone, in front of the sterno-mastoid. It was, however, soft; on percussion it gave a tympanitic percussion note, and it was clearly anchored to the top of the larynx, the movements of which it followed during deglutition. Pressure on the tumour led to an obvious diminution in its size, produced a squeaking sound, and at the same time caused pain extending to the ear which persisted for some days. Removal of the pouch was advised, but the patient could not make up his mind and he was not seen again until December 31, when he was admitted to University College Hospital. The history he gave was interesting. He was 31 years of age. Twelve years ago he had received a blow from a fist on the left side of the neck, which, although painful, was followed by no special symptoms at the time. Two years after this he began to feel something working up and down on the left side of the neck on swallowing, and during the last three years he had been liable to acute attacks, lasting usually about a fortnight, during which a lump developed on the left side of the neck, and swallowing became increasingly difficult and painful until he could only swallow fluids. The last attack had been much worse than usual, and for the first time the skin over the swelling had become red. On admission the tumour was very painful, and reached the middle line in front, and the posterior border of the sterno-mastoid behind, overlapping and pressing back this muscle. The tumour was freely movable over the deeper parts except near the upper part of the larynx to which it was fixed, the carotid vessels passed behind it. The percussion note all over the tumour was tympanitic, and he could slightly inflate it by holding his breath and blowing. The pouch was removed on January 2. The dissection required was rather a delicate matter. The upper part of the pouch was found to consist of two portions communicating by a rather small opening, but when the narrow pedicle was reached, which passed through the thyro-hyoid membrane, careful probing failed to discover the actual communication with the pharynx. The tubular pedicle was secured bj catgut stitches, a portion of it being divided as each stitch was passed, and finally the stump was invaginated by four stitches passed after the manner of Lembert's sutures. The wound healed perfectly. About 200 cases of pharyngeal pouch and fistula have been recorded. They probably all arise in connection with one or other of the visceral clefts, and the following varieties were given by the authors of this paper as depending upon the position of the obliteration of the cleft: (1) it may remain open throughout its entire length, giving rise to the complete fistula opening into the pharynx and on the side of the neck; (2) no external opening may be formed and a pharyngeal pouch with a blind extremity may remain; (3) the internal aperture may become shut off from the pharynx, leaving a blind external fistula; and (4) both internal and external openings being closed a "dermoid" cyst of the neck may remain. In more than a third of all the cases the fistula was complete, and of the remainder the majority presented an external fistula, either blind or with the internal orifice so concealed that it could not be found. It is at least probable that some of the so-called pressure pouches of the œsophagus originate from pouches which may be placed in the same category as the above.

